# A qualitative study into the experiences of families affected by developmental disorders seeing the UK NHS Genetics Service; if I had known then what I know now

**DOI:** 10.1002/jgc4.70063

**Published:** 2025-06-03

**Authors:** Emma Carter, Flora Joseph

**Affiliations:** ^1^ Centre for Medical Education, School of Medicine Cardiff University Cardiff UK

**Keywords:** disability, family, Genetics Service

## Abstract

Parents of a child with a developmental disorder (DD) experience significant challenges, such as prognostic uncertainty, lack of care coordination, stigmatization, and changes to social and financial positions. Limited research exists into whether parents' support needs are being met by the United Kingdom National Health Service (UK NHS) Genetics Service. Therefore, this study aimed to establish whether these parents feel adequately supported by the UK NHS Genetics Service and, if not, what further support could be provided. This study recruited participants through the Unique and SWAN UK support groups. Fourteen parents of children with a DD took part in semi‐structured interviews. Four overarching themes were identified: Expectations, the impact of the delivery of the diagnosis, uncertainty about who has medical responsibility, and isolation. While some positive experiences were described, parents also revealed expectations of support from the Genetics Service that were not met. These expectations included support with care coordination, a medical professional to take a holistic approach, and being signposted effectively to support networks. The analysis suggests that patient expectations of the Genetics Service need to be managed prior to the first appointment and that parents would benefit from access to a dedicated care coordinator. Furthermore, signposting to support groups is inconsistent. Future research should focus on identifying families most in need of support so that these families can be prioritized for the limited resources and investigate how best to prepare patients for receiving a diagnosis.

**What is known about this topic**

Parents of children with a DD face many challenges, such as a lack of information, emotional and psychological distress, and social stigma, which can negatively impact their mental well‐being. The genetics service is critical in providing families the much‐needed support.

**What this paper adds to the topic**

This study identified that the expectations and support needs of some parents of children with a DD are not being met by the UK NHS Genetics Service or other healthcare services. This paper explores this and discusses clinical implications to help address these unmet expectations and support needs.

## 1 INTRODUCTION

Developmental disorders (DDs) form the largest group of child disabilities, with a reported prevalence of 3%–4% of all children in the United Kingdom (Emerson, 2012). DDs comprise a group of conditions in which a person's learning, memory, or application of certain skills and information are affected (Sulkes, 2020). Parents of children with a DD are usually the primary caregivers of the child.

It is widely acknowledged that parents of a child with a disability experience more stressors throughout their life than parents of a child without a disability. This is partly due to the additional, unique health and social care needs that a developmental disability (DD) can bring and the impacts these have (Hsiao, 2018; Kandel & Merrick, 2007; McConnell & Savage, 2015). Having a child with a DD can have many psychosocial and emotional implications on parents and families, including changes to social support, financial position, family dynamics, and their mental well‐being (Kandel & Merrick, 2007). Some studies show that parents of a child with a DD have increased rates of depression, anxiety, and stress compared to parents of typically developing children (Kandel & Merrick, 2007; Olsson & Hwang, 2001; Scherer et al., 2019).

However, some studies report that despite the stressors that come with having a child with a DD, families are able to manage life effectively and experience positive outcomes such as successful adaptation and coping mechanisms (Beighton & Wills, 2019; Emerson, 2003; Kandel & Merrick, 2007; Scorgie & Sobsey, 2004).

Parents of children with a DD often face many barriers and challenges in the health and social care setting, such as stigmatization and lack of access to knowledge and expertise (Mitter et al., 2018; Rare Disease, 2016; Song et al., 2018). Stigmatization has been associated with negative implications including chronic stress and poor health outcomes in parents of a child with a DD (Song et al., 2018). Parents of a child with a DD also face a lack of information available regarding their child's condition. Up to 50% of children with a DD remain genetically undiagnosed (Rare Disease, 2016), and even for those that do have a diagnosis, the condition can be so rare that little information is known. Prognostic uncertainty can have negative impacts on mental and physical well‐being, including feelings of stress, worry, anxiety, and lack of perceived control, optimism, coping, and adaptation (Aldiss et al., 2021; Inglese et al., 2019; Madeo et al., 2012; Rare Disease UK, 2016).

In addition to the lack of specific knowledge available for parents, some patients with DDs are not under specialist professionals or centers, but rather community primary care clinicians such as the general practitioner (GP; a consultant in general practice) or the pediatrician (Aldiss et al., 2021). This lack of overall care coordination could result in parents of children with a DD feeling anxious, uncertain, confused, and alone with this medical responsibility (Oulton et al., 2020).

The UK NHS is the UK's publicly funded healthcare system. The role of the UK NHS Clinical Genetics Service is to try and find an underlying cause for a likely genetic condition. Genetic clinicians strive to give families tailored genetic information and answer questions related to the genetic diagnosis, as well as psychological and emotional support, to help minimize psychological distress, negative feelings, and feelings of uncertainty (Bisecker, 2002). Appointments are typically 30–60 min in length and may be with Clinical Geneticists and/or Genetic Counselors. While Clinical Geneticists may offer several appointments and guide the management decisions for a condition, they generally are not the professionals who provide therapeutic counseling or continue patient care management. Rather, they make referrals to other disciplines for this, such as Community pediatrics (NHS commissioning board, 2013).

Mainstream clinicians in the United Kingdom, such as pediatricians, can now order some genetic tests and give results of their requested test to patients. Patients are often referred to Clinical Geneticists at this point to further discuss the genetic variant identified, cascade testing, reproductive options, or further diagnostic genetic testing. Genetic test requests and acceptance of referrals to the Genetics Service are subject to service specifications and the national test directory criteria (National Genomic Test Directory, 2025; NHS Commissioning Board, 2013).

Traditionally, genetics professionals in the United Kingdom have had the time and resources to give patients/families long‐term support and follow‐up (Skirton et al., 1997); however, increased pressures have resulted in the UK NHS Genetics Service becoming time‐sensitive and limited in its ability to offer long‐term counseling (Benjamin et al., 2020; Cohen et al., 2012; Wiggins & Middleton, 2013). Furthermore, the advances in technology i.e. whole genome sequencing (WGS) have also led to a greater number of novel diagnoses and increased demand for genetic testing (Inglese et al., 2019). Often, little is known about these very rare conditions, and due to an increased demand for services, an increased number of parents are at risk of feeling unsupported and uncertain about their child's condition despite receiving a diagnosis (Joseph, 2019).

Parents of children with a genetic disorder in the United Kingdom have described their diagnostic sessions with medical genetics as negative, partly attributable to the lack of emotional support and information, and were left with feelings of uncertainty and the need for greater social support (Ashtiani et al., 2014; Inglese et al., 2019; Rare Disease, 2016).

However, these studies were not specific to DDs and responses were limited as to whether this extra support is expected from the UK NHS Genetics Service specifically, and if so, what further support is needed. Inadequate support can lead to a person feeling isolated, helpless, and out of control, which can have negative effects on a person's mental health and personal development (Cauda‐Laufer, 2017). This study aimed to investigate whether parents of a child with a DD feel in need of further support from the UK NHS Genetics Service and, if so, what additional support do they feel most in need of.

## 2 METHODOLOGY

### 2.1 Study design

This study used an interpretivist framework to make meaning of the lived experience of parents/guardians of a child with a DD regarding feeling adequately supported by the UK NHS Genetics Service. A qualitative approach, utilizing semi‐structured interviews, was chosen for this study due to its explorative nature.

### 2.2 Participants

This study's target sample was parents/guardians of a child with a DD, who spoke fluent English and who had had an appointment with a UK NHS Genetics Service for their child within the last 10 years. This sample was thought to have the most relevant experiences and perceptions to provide sufficient informational power to fulfill the aims of the study and be useful for future service planning, compared with patients who had not been seen by the Genetics Service or had been seen longer than 10 years ago due to changes in genetic testing and processes within Genetics Services (Horton & Lucassen, 2019; Malterud et al., 2016).

To gain access to this target sample, purposive sampling methods were used. This study aimed to recruit 12–14 participants overall, 6–7 participants from both SWAN UK and Unique. In addition to sample selectivity, the authors considered Malterud et al.'s (2016) four other proposed principles of power, study aim, established theory, dialogue quality and analysis strategy, and deemed this sample size to give sufficient informational power to fulfill the study's aim, as well as being achievable and manageable (Braun & Clarke, 2013, 2024; Fugard & Potts, 2015; Malterud et al., 2016).

Participants were recruited through the support groups SWAN UK and Unique. SWAN UK and Unique are both well‐established registered UK‐based charities. SWAN UK provides support for families affected by an undiagnosed “syndrome without a name,” and Unique provides support for families affected by rare chromosome and gene disorders. Potential participants who were members of Unique were recruited through the Unique closed Facebook page. The recruitment post, posted by their administrative team, had attached a participant information sheet and a consent form. People who wished to take part in the study then either contacted the student researcher with questions or sent the consent form to the student researcher via email. Potential participants who were members of SWAN were recruited through a post, posted by their administrative team, on the SWAN UK closed Facebook group and X, formerly known as Twitter. People who wished to take part in the study then contacted the student researcher via email. The student researcher then sent the participant information sheet and the consent form to the potential participant. Once potential participants from SWAN or Unique contacted the student researcher, the researcher contacted the participants directly to answer any questions, obtain consent if not already obtained, and arrange a date, time, and telephone number for the interview. Participants had the opportunity to ask any questions via email or request a call to discuss any questions at any point.

### 2.3 Data generation

Individual telephone interviews were conducted by the first author (EC), who had received basic training in interview techniques through the Cardiff University Genetic Counseling Masters course, due to the geographical dispersion of participants. EC read the research question, the aims of the study, and the role of the UK NHS Genetics Service before starting with the questions. The semi‐structured interview guide (Appendix A in Data S1) consisted of 22 questions: three demographic questions, nine closed questions, and 10 open‐ended questions. This guide was developed based on a review of the literature to facilitate conversations to help fulfill the aims of this study and fill gaps in the existing literature. Interviews were audio recorded, with permission from participants, and transcribed verbatim post‐interview by EC. Transcripts were anonymized and numbered, and pseudonyms were used for confidentiality purposes.

### 2.4 Data analysis

Interview data was subjected to reflexive thematic analysis as outlined by Braun and Clarke (2006, 2013, 2024); the interviews were analyzed as quickly as possible after the interview had taken place. Codes were applied to the data, using descriptive and in vivo coding techniques, by picking out interesting features, which were then organized and grouped into relevant preliminary semantic themes (Saldaña, 2009). A theme represents a common thought or meaning within the data that relates to the research question, and the theme name captures the “essence” of that theme (Braun & Clarke, 2006). Analysis occurred throughout the data collection phase so that identified codes and preliminary themes could inform following interviews. Codes and themes were modified throughout the analysis process, allowing emergent features to be incorporated as more interview transcripts were analyzed (Braun & Clarke, 2024).

All transcripts were read numerous times to enable familiarization with the dataset. Themes were reviewed, refined, and named, and a thematic map was produced to aid in showing how themes link together, provide clear analysis of the dataset as a whole, and offer insight into how the data fits with the research question. The thematic map is not included in this publication and was only used internally by the research team. The second author, Flora Joseph (FJ), also became familiar with several of the transcripts and reviewed and edited the codes and themes. This allowed for a collaborative analysis and expanded the perspectives of the researchers, beneficial in an interpretivist paradigm.

Reflexive thematic analysis does not require categories, algorithms, or hypotheses to be thought of prior to the interviews; rather, it is reflexive and organic. This was appropriate and advantageous for this study due to this study's exploratory nature and therefore the need for the analysis to have an inductive approach. Furthermore, this analysis enabled perceptions and thoughts to be presented in a systematic way while retaining closeness to the raw data, which is appropriate due to the subjective nature of qualitative research (Aldiss et al., 2021; Nowell et al., 2017).

## 3 POSITIONALITY STATEMENT

The authors are a UK‐registered genetic counselor and a MSc Genetic Counseling student. This project was the basis for EC's dissertation for her Master's degree. The question for this project was raised by FJ while working in an NHS Clinical Genetics Service and observing practice around the United Kingdom over 12 years. EC's interest in this project stemmed from her own experiences with families with DDs in a care setting, and then within the genetics department as a genomic associate. This experience led to an interest in the healthcare and support families affected by a DD received and required, and a desire to help families be heard by the Genetics Service and instigate positive change. FJ had previously published an opinion piece in 2019 about parents of a child with a DD and their experiences of the UK NHS Genetics Service. The hopes for this project were to ascertain whether there was a support gap and whether the UK NHS Genetics Service was meeting the expectations of families that they served within this patient group. Both researchers are of white British ethnicity and of middle‐class background. The authors had clear roles within the team outlined prior to starting the research. It is hoped, after publication, this work can be delivered to UK NHS Genetics Service via teaching sessions and to the patient groups via the charities themselves.

The authors recognize that their backgrounds and experiences influenced their interpretation of the data in this interpretivist methodological paradigm. EC and FJ discussed their backgrounds throughout data analysis to increase awareness of their positionalities.

## 4 ANALYSIS

### 4.1 Recruitment

**FIGURE 1:**
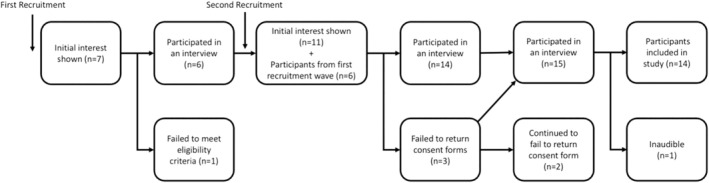
The number of participants enrolling into the study throughout the recruitment process. EC received seven enquiries following the initial advertisement in November 2022, out of which one potential participant did not meet the eligibility criteria. The remaining six potential participants took part in this study. The recruitment post was advertised again in January 2023. EC received 11 responses, all of whom met the eligibility criteria. Out of these 11 potential participants, eight potential participants went on to take part in this study straightaway. Three failed to respond to EC with the consent form and arrange a time for the interview. After 1 month, EC sent a follow‐up email. Two potential participants continued to not respond, while one potential participant responded and went on to take part in the study. One interview was not audible and was not included in the analysis. This left 14 participants' interview data being included in data analysis, 10 of whom were Unique members and four of whom were SWAN UK members.

The number of participants enrolling into this study throughout the recruitment process is summarized in Figure 1. In total, 14 participants' interview data were included in data analysis, 10 of whom were Unique members and four of whom were SWAN UK members. This sample size met the study's aim for recruitment. As the number of Unique and SWAN UK members that met the study's eligibility criteria is not known, a response rate could not be calculated. Interviews lasted between 17 and 45 min.

### 4.2 Sample demographics

The characteristics of the 14 anonymized participants are shown in Table 1.

**TABLE 1:** Sample demographics and information.

Demographic	Category	Number
Sex	Male	2
	Female	12
Support group	SWAN	4
	UNIQUE	10
Level of Education	No qualifications	1
	High school qualifications or equivalent	0
	College/Sixth form qualifications or equivalent	3
	Bachelor's degree	7
	Post‐graduate degree	3
Age of child	0–3	8
	3–6	2
	6–9	2
	9+	2
Age of participant	26–30	1
	31–35	4
	36–40	3
	41–45	5
	46–50	1
Regional genetics service	South‐east Scotland	1
	Yorkshire	1
	Manchester	3
	Wales	3
	East Anglia	1
	London (North east Thames, South East Thames, South west Thames)	4
	Wessex	3

*Note*: The 14 participants' demographics are shown, including gender, age of participant, and their child with a DD at the time of being seen by the Genetics Service, their support group, their level of education at the time of being seen by the Genetics Service and the Regional Genetics Service attended. One participant's child had been seen at several different Genetics Services. All participants' ethnicity was White British.

### 4.3 Analysis and themes

Thematic analysis was carried out. The authors generated four main overarching themes: Hopes and Expectations, the day the diagnosis was given, medical responsibility, and isolation.

#### 4.3.1 Theme one: Hopes and expectations

This theme relates to parents' expectations of being referred to the Genetics Service when it was appropriate, and the role of the Genetics Service prior to first being seen.

##### A genetics referral will be made when appropriate

The expectation was that a referral would be made when it was indicated. However, it became clear that this was not always a straightforward process for families, with two parents referring to accessing the Genetics Service specifically as a “fight.”

We have to fight tooth and nail to get [a referral to the Genetics Service]. Which is also upsetting along with the whole process of erm, you know, you don't want to feel like you have to beg anybody. pp14

Some parents believed that this challenge in getting a referral was partly attributable to healthcare professionals lacking knowledge of genetics and rare genetic conditions. Parents suggested more training is needed for primary healthcare professionals.

I find the kind of idea that people, paediatrics are the gatekeeper to genetics is something that you know paediatricians need more training in. pp3

It became clear throughout the interviews that this “fight” for a referral was not limited to the Genetics Service. Seven parents experienced difficulties accessing many other services too, such as medical referrals, welfare benefits, and educational assessments. Parents described these constant fights for services as being exhausting, frustrating, and time‐consuming. Some felt that the lack of a clear pathway for children with a DD contributed to them having to fight for every referral as there are no guidelines for primary care clinicians to follow.

I was fighting for her to be statemented at school with everything, with education, with medical things, with everything… you just go round in circles trying to fight everybody and scream as loud as you can until somebody hears you and does… something to help. pp2

This time‐consuming fight seemed to have cost four parents financially as they felt they had to pay for some services privately or give up their jobs to dedicate time to navigating the healthcare service and to fight for referrals.

I actually took redundancy [Redundancy is a form of dismissal from your employment. This occurs when employers need to reduce their workforce] cause I couldn't manage keeping on top of all of that… But obviously that's had a massive impact on us as a family. pp7

Some participants gave smooth, positive referral stories, demonstrating variation in provision across the United Kingdom. However, for some of these parents, a genetic diagnosis had already been identified through investigations arranged by their pediatrician, and it was this diagnosis that led to the referral to the Genetics Service.

Overall, this theme was constructed as many participants did not know how to navigate the system (accessing the Genetics Services or other health and social care services) and suffered negatively because of this.

##### Expectations of the role of the Genetics Service

Twelve of 14 participants reported that their expectations had not been met by the UK NHS Genetics Service. Prior to their genetics appointment, parents anticipated that the Genetics Service would be able to provide long‐term follow‐up and care coordination for their child, therapeutic counseling, and/or information regarding the underlying cause of their child's disability, their child's future progression and outcomes, and implications for family members.

I thought they would have more time for us and just generally give us more time and explain stuff and allow us to come back to them. pp9

Participants felt inadequately prepared for their genetics appointment, either not knowing what to expect or having misinformed expectations. This often led to parents having unmet expectations, which had negative impacts.

#### 4.3.2 Theme two: The day the diagnosis was given

The second theme created, revolves around receiving the diagnosis itself, both the manner of receiving the diagnosis and the impact this had on parents, as this became an important part of the family's story.

##### Method of receiving the diagnosis

The interviews demonstrated that the method by which a parent received their child's diagnosis impacted their experience. Three parents had been seen by and given the result by a Clinical Geneticist, while seven parents had been seen by and given the result by a mainstream clinician.

The majority of parents (4/7) who had been given the diagnosis by the mainstream clinician explained how they felt that they should not have been given the result by the mainstream clinician due to their lack of understanding and knowledge. Furthermore, parents explained that the wait between being given the diagnosis by the mainstream clinician and being seen by the Geneticist had felt too long, which had further negatively impacted them emotionally.

Well, we didn't receive our diagnosis through the genetics department, which I think probably wasn't the best. We received it through the [healthcare professional], so he didn't give it in, probably, the most appropriate way. But when the genetics team then met us a few months later, that meeting was really lovely…. But yeah, that kind of first point of contact, that dissemination of information was really poor. Looking back now, that first kind of six months, it was pretty awful. pp11

In contrast, two parents felt little need for an appointment with the Genetics Service following their results appointment with the mainstream clinician. This was because the parents felt that the Geneticist was not able to provide any additional information to their mainstream clinician and their own research. However, one of these parents had been highly educated in a scientific field.

On the other hand, feelings of parents who received the diagnosis from the Geneticists were mixed. Some parents were happy with the Geneticist, perceiving them to be sympathetic, helpful, and lovely in nature.

Good really. You know to start with explaining everything. You know… er… good positive interactions… It was good the whole way it was just good from the start. pp5

While others perceived the Geneticist to be lacking in emotional understanding, reporting that clinicians did not seem to understand how life‐changing receiving the diagnosis was to them, and that for the clinician, reporting a diagnosis was just a day‐to‐day occurrence.

sometimes professionals forget how life‐changing the news is… they just do it all day every day and they kind of don't realise the impact of what they're saying on, like, the family. pp9

Three parents reported how the COVID‐19 pandemic negatively impacted them when receiving their child's diagnosis. Two parents discussed how the nature of the appointment being on the telephone was not appropriate and how they would have preferred a face‐to‐face appointment. This had been distressing for one parent to think back to.

Another parent explained that their partner was not allowed into the appointment, which was incredibly difficult emotionally, and that they felt responsible for reporting the information back to their partner and felt that it should not have been their responsibility to do.

I don't know how they would be if it wasn't the height of Covid, but I think sometimes, especially when giving a diagnosis, you should allow both parents in the room. You know, because it's mean not to, basically I had no support in that appointment. pp9

Overall, the method of how participants received their diagnosis influenced how supported they felt and the amount of additional support they felt in need of. In general, those receiving the diagnosis from the mainstream clinician felt most in need of additional support initially after receiving the result, except for two exceptions. Furthermore, the nature of a results appointment, that being in person and having a support person present, improved the overall experience for participants.

##### Emotional impact of receiving a diagnosis

Parents discussed in depth the emotional implications that receiving a genetic diagnosis had on them. Many parents repeatedly referred to receiving the diagnosis as “traumatic” and a “shock.” This was the case for both those who had been given the result by the mainstream clinician and the Geneticist. Six parents discussed how they were unprepared to receive the diagnosis with two parents stating that they never really expected to receive a diagnosis. A warning prior to the appointment was suggested as a necessary change to reduce the shock and trauma felt and that this would have enabled them to be in the right mindset to take in new information and also encourage them to bring someone who could support them and help take care of their child during the appointment so they could focus on the new information.

So, I was a bit traumatised when I came out. I didn't really know what to do … it's shock more than anything. And then you think cause you're in shock, you're not really taking on board what they're saying to you. You could just do with having five minutes and then coming back and saying, right… There was no mention that it gonna go to be any different. And I really wish now that I'd brought somebody else could've come with me so that I could've actually listen to what she was saying. pp1

Additionally, parents commented on how the difficulty of receiving the diagnosis was heightened by the lack of information available regarding their diagnosis. Parents expected more information from the Genetics Service and felt unprepared for the little information that was provided and the uncertainty. Overall, 13 out of 14 parents felt that they received a lack of information.

And the answer was, come back to us in 20 years perhaps, we don't know. And that was it really…. We had some questions and obviously they couldn't answer them really. And that was it. We were sent on our way. pp4

I took six months out on stress, and I ended up having counselling cause I couldn't cope with the uncertainty. pp7

One parent described how, although the lack of information and uncertainty is difficult with a diagnosis, not having a diagnosis is worse. Whereas another parent felt the opposite, saying that it is worse when one has a diagnosis.

Four parents perceived the laboratory report to be a vital piece of information. Often, parents were not given this report in their appointment, which seemed to be a common reason parents got back in touch with the Genetics Service. Parents indicated that they would have liked this report at the point of receiving the diagnosis, as this would have meant that they had the name of the diagnosis in writing when leaving the appointment.

This theme shows that receiving the diagnosis can be a significant event for parents and that having further support in place around this could reduce parents' potential negative feelings associated with this.

#### 4.3.3 Theme three: Uncertainty about who has medical responsibility

Parents still hoping for a diagnosis, and those who had received one, frequently explored how they felt after seeing the Genetics Service, reflecting back on their experiences, thoughts, and emotions. There was apparent uncertainty about where the medical responsibility lay, and many felt it fell to them.

Two out of the four (50%) parents of children without a diagnosis seemed to feel responsible for having to notice clinical features that would lead to a diagnosis or would enable their child to meet the restricted testing criteria for further genetic testing. This approach led to parents feeling under pressure, anxious, and sometimes medicalizing their child. One parent in particular was very frustrated at the restricted testing criteria as they wanted to put things in place before the features presented in their child, and they expressed how sometimes exceptions needed to be made, especially when failing to test has an extremely negative impact on parents' mental well‐being.

It's sort of stabbing around in the dark … And you are just left to kind of Google and worry and stare at your child wondering if there's something you're missing… We're sort of just watching a child and every time she has a bath or every time she's running or doing something, you're just looking at like what I'm looking out for … it's just a constant at the back of your mind… I sort of, I feel like we've just been batted away. pp2

Nine out of 10 parents who had received a genetic diagnosis for their child felt left alone to deal with the diagnosis. Parents described that as soon as the Genetics Service had found the diagnosis, the support from the Genetics Service immediately stopped. The one parent who did not feel this had three appointments with the Genetics Service in addition to the appointment with the mainstream clinician when they received the diagnosis.

Cause anything I've learned, I've had to learn myself on the internet. And you're never quite sure if that's correct… But I did feel bit like we found the problem bye, see you later, don't come back cause we can't help you, don't come to us… all of a sudden, you're left to be a genetic consultant yourself. pp1

Several parents expressed the desire for a follow‐up appointment with the Genetics Service to be able to ask questions once the initial shock had subsided. Although most parents were told to get in touch with the Genetics Service if they needed to, three parents felt that they did not know how to, and four parents felt that although they knew how to, getting in contact was a challenging process.

Two parents felt that they got support from other healthcare professionals in other departments, which was vital to them in understanding the genetic diagnosis. Parents recognized that without these other medical professionals going out of their way and beyond their job role to help, they would have felt very lost and confused.

A very lucky experience with the [other healthcare professional] with me. But I don't know what I would have done really if I hadn't had him really. pp14

On the other hand, two parents felt satisfied with how things were left by the Genetics Service, and were aware of how to get back in touch with the department if and when they needed. Furthermore, those who had received the diagnosis by the mainstream clinician and then saw genetics clinican seemed more pleased with how things were left than those who received the diagnosis from the Genetics Service.

yeah, since then we saw the Genetics Consultant then I think twice then after that, saw him after and then we saw him for a follow up the following year. Both times he was really helpful, really interested and making sure that we had the right people involved in his care and the right referrals in place. pp11

This suggests that those that have received the diagnosis prior to seeing Genetics have had time to assimilate their thoughts and questions. In this regard, the appointment with Genetics acts like a follow‐up appointment, as indicated by the parents who received the diagnosis from Genetics, which they would have found useful.

In contrast, the majority of parents of a child without a diagnosis did not express these feelings of abandonment in terms of follow‐up. In fact, one parent perceived having received an ongoing commitment from the Genetics Service in helping them find an explanation for their child's disability. Furthermore, parents of children without a diagnosis largely had more appointments with the Genetics Service than those of children with a diagnosis.

[Regional Genetics Service] have said that they won't drop us until they find an answer. Even if the NHS money runs out, they'll fund the research. pp2

Again, this shows that parents feel in need of additional follow‐up support, especially post‐diagnosis once the shock of the diagnosis has passed.

##### Lack of specific care coordination

Parents described that although their child might be referred to and seen by individual professionals for a period of time for a specific health concern, there was no clear pathway or a specific health professional overseeing their child's care and taking responsibility for their child's overall health. This left parents feeling solely responsible for their child's medical needs and appointments. This was felt by parents of children with and without a genetic diagnosis.

It falls apart. You get thrown out of genetics you know, not diagnosed or you are diagnosed, and you know, and suddenly you'll be put back into the community and the community's completely un‐joined up… there isn't actually a system or a path you get on. pp3

Twelve parents also discussed the struggles in coordinating their child's appointments. Parents also felt responsible for passing medical information between healthcare professionals and facilitating their conversations, despite not having a medical background. Parents commented on the practical, emotional, and financial strains this had put on their family. All parents expressed great interest in having a clinic coordinator.

Bain of my life [care coordination]. I tried talking about it at multi‐disciplinary teams, but half of the f***ing healthcare professionals don't turn up. Yeah, but we're under … four different hospitals, I think under 12, 14 consultants now. It's ridiculous. It's insane. And they don't talk to each other and there's absolutely no form of coordination and it drives me nutty. pp9

It was apparent to the authors that many parents do not feel adequately supported when coordinating their child's care and in need of a professional to help coordinate their child's care and take responsibility.

#### 4.3.4 Theme 4: Isolation

Parents discussed their support outside of the Genetics Service and the impact positive support had had on them and their family. However, parents still felt in need of additional support from a healthcare professional. This support fell into two categories, “Family and friends,” and “support groups.” We also discuss the occurrence of appropriate signposting in our study cohort.

##### Family and friends

Eleven parents discussed how family and friends (excluding spouses/partners) were not able to support them regarding their child's condition as they could not understand what they were going through and often also encouraged a “wait and see” approach. This often frustrated parents and made them feel alone and unsupported.

My partner was supportive but not really family because up to the diagnosis nobody really knew what was wrong, and probably people didn't think it was as bad as it actually was. pp1

Two parents reported that this changed with a diagnosis as family and friends were able to begin to understand that there were difficulties and then support them emotionally and practically. However, a diagnosis did not always result in an increase in understanding, with some parents feeling that their friends or family could not understand the diagnosis due to the condition being so rare.

I think it's really hard for everyone else to understand, because it's such a rare condition … so I just don't think a lot of people understood exactly what was wrong. pp10

##### Support groups

The majority of parents praised their support group (Unique or SWAN UK) for their ability to provide information, answer questions, and connect them with families with children with the same or similar condition or with families in their local area who could also provide information and support.

We found the only information we could get was from Unique… If they [Unique] as a first port of call weren't there, I don't know where we would be now. pp7

Parents also described how Unique had given them the ability to access specific groups with parents of children who had their child's specific genetic diagnosis. Parents deemed these groups to be very important in getting them through difficult times and also beneficial in helping their child get the best care possible as the small groups allowed them to collect medical knowledge from around the world such as suitable medication.

I've kind of created a little group, just a space for parents to ask each other questions. … And I used it. So, our daughter got diagnosed with condition in [time of year] and I used it with the … consultant at the hospital to help narrow down what medication to start on because I could ask all the other parents who'd got the same condition what medication they were taking which was amazing. pp7

Despite the recognition of this support, the support group usually did not stop parents perceived need for additional support from the Genetics Service, with the majority of parents answering “no” to “If you feel all your needs were not addressed, were they addressed by others e.g. other healthcare specialties, a support group, friends/family – if so, who?” Parents discussed how support groups helped address some needs such as providing trustworthy and understandable information, and connecting them with other families. However, the support groups did not fully satisfy many parents perceived need for follow‐up from a medical professional and care coordination.

Therefore, despite the positive experiences' parents had of support groups in relation to information provision and social support, their support groups could not fill all the unmet support needs of parents of a child with a DD.

##### Signposting

Although three parents had been given information on patient support groups (signposted) by their clinician or Geneticist, the vast majority (11/14) had not been signposted to any support groups; rather, they had found their support group themselves, which had sometimes taken years to find. Our study included parents' experiences of nine different Genetics Services in the United Kingdom, thus indicating this to be a national issue. Parents were confused and struggled to understand why they had not been informed about available support groups and that going forward, this is something that needs to change.

It's just shocking that you know even for like SWAN, I wasn't given any information, any leaflets. Nobody's erm, I've got a whole folder, I've kept everything, every letter, every appointment letter I've got nothing… And I only found SWAN two years ago. That's seven years of not knowing that there was this thing out there… That could have been a support which I think is really bad. pp2

Social media also seems to have facilitated many parents to connect with other families, gain insights into challenges for other families, find information, and to find other support groups such as SWAN UK and Unique. Social media appeared to be a significant source of support for parents.

We were lucky to find a support group on Facebook for her condition, which actually gave us so much more information. pp4

This shows that there is a lack of signposting to support groups and shows the negative implications this had on parents, thus directly showing how parents would like additional support from the Genetics Service. Furthermore, a lack of signposting to support groups may also increase parents' need for further support by the Genetics Service.

## 5 DISCUSSION

This study found that parents of children with a DD have misconstrued expectations of the UK NHS Genetics Service and provided areas where the Genetics Service could address their support needs better.

Expectation refers to a person's belief about what will happen in a future situation (Lateef, 2011). In healthcare, patient expectations have been found to impact patient satisfaction, a valued patient outcome of the NHS (McKinley et al., 2002). Despite this, little research is available regarding whether the expectations of parents of a child with a DD have of the UK NHS Genetics Service are appropriate and whether they are being met or not. This study found that parents had several unmet expectations, particularly in relation to the amount of information received and ongoing support. These findings suggest some areas where the UK NHS Genetics Service could improve, but also how some expectations are misaligned with the true role of the UK NHS Genetics Service.

This study found that many participants expected that the UK NHS Genetics Service was going to be able to provide relevant information, answers, and explanations regarding their child's condition. However, the participants of this study found that this expectation was rarely met by their Genetics Service. In part, this is due to the limitations in the ability of making a genetic diagnosis, but also about the lack of information about recently described conditions. This has previously been highlighted by other studies to be a difficult issue for parents of children without a genetic diagnosis (Madeo et al., 2012) and with a genetic diagnosis (Rare Disease, 2016). There remains a mismatch between parental expectations and reality.

A lack of information has been found to lead to increased feelings of uncertainty and anxiety, and reduced parental coping, adjustment, and perceived control (Garrino et al., 2015; Madeo et al., 2012; Von der Lippe et al., 2017). Our study suggests that these outcomes can be further negatively impacted if parents had initially been expecting to receive information. This is because unmet expectations have also been associated with negative emotions including shock, disappointment, anger, and dissatisfaction (Jackson & Kroenke's, 2001; McKinley et al., 2002). This suggests that the emotional implications and outcomes of a combination of unmet expectations and the lack of information could be much worse than the lack of information alone.

Therefore, parents' expectations, in relation to information provision by the Genetics Service, may need to be proactively managed prior to, or during, the first genetics appointment. Although it would not prevent emotions associated with a lack of information and uncertainty, this could reduce the aforementioned negative emotions associated with unmet expectations and also allow parents to prepare for dealing with the continued uncertainty.

Another area in which parents' expectations were not met was the amount of follow‐up, counseling, and on‐going medical oversight for their child that they received from the UK NHS Genetics Service, which left parents feeling abandoned and in need of additional support for themselves and their child. This also suggests a misunderstanding by referring clinicians about therapeutic counseling provision by Clinical Geneticists, which is consistent with previous research studies (Delikurt et al., 2015).

We propose three possible explanations for why parents felt medically abandoned by the UK NHS Genetics Service and in need of further support.

Parents held an initial expectation that they would be guided or follow a care pathway, but due to the nature of undiagnosed or very rare conditions, there is often no care pathway or condition‐specific health professional. There was a lack of clarity about who held the responsibility for care coordination. While the pediatrician would have the overarching responsibility for monitoring development, Clinical Geneticists may be considered to be the experts in very rare conditions, and that ongoing care coordination could come from there. However, as Clinical Genetics is predominantly a diagnostic specialty only, this mantle is not picked up and therefore parents are left to be the care coordinators for their child. This led to most parents feeling that they have no other professional to turn to who could answer their questions or listen to their concerns. This was in line with the findings of Currie and Szabo (2019) who similarly interviewed 15 parents (11 mothers, 4 fathers) of children with a genetic diagnosis of a neurodevelopmental disorder.

Second, a lack of signposting to support groups may also increase parents' need for further support from the UK NHS Genetics Service. Accessing social support and support groups has been shown to be an emotional coping strategy, helping to increase perceived control, adaptation, and optimism (Bromley et al., 2004; Lipinski et al., 2006). However, we found that approximately 80% of participants were not signposted to Unique or SWAN UK by the Genetics Service, which is consistent with the findings from the Rare Disease (2016) survey, which found that 80% of participants were not signposted to a support group at the time of diagnosis, increasing the reliability of our findings. Therefore, we suggest that signposting parents and patients to a support group may need to become a standard practice to reduce the identified negative implications for parents if not signposted.

Third, parents can be either stressed or in shock during their genetics appointments. Shock, stress, and trauma have been associated with a reduced ability to process and retain information (Vogel & Schwabe, 2016), thus potentially resulting in parents forgetting information and not utilizing appointments to ask questions. This could be a contributing factor in explaining why parents in the current study felt in need of additional follow‐up. A “warning” prior to receiving the diagnosis could help to reduce this shock and encourage patients to prepare for the appointment. This may in turn reduce parents perceiving a need for follow‐up appointments.

Despite these unmet expectations of parents by the UK NHS Genetics Service, many spoke positively regarding their interactions with their Geneticist and understood that the service was overstretched and that the lack of information, although disappointing, was not their clinician's fault but was primarily due to the lack of publications about their child's syndrome. We found that parents did not feel rushed in their genetics appointments and had positive interactions with their Geneticist. However, this did not eliminate the desire and need for follow‐up appointments and additional support.

Previous studies have found that having a child with a DD often leads to changes in social support (Kandel & Merrick, 2007; McConnell & Savage, 2015). We found that the majority of parents stated that family and friends were often unable to support them regarding their child.

The internet, social media, and support groups were often given as sources of information and support for parents of children with a DD. Despite a recent increase in funding and research into rare diseases (The Department of Health and Social Care, 2023), parents still find that the information available to them is limited and is often either too generic (Julie McMullan et al., 2020) or too specific and technical (Rare Disease, 2016). Parents in our study commented that research papers were often not relevant to their child and were also heavily filled with medical jargon that was difficult to understand and interpret. Parents reported that information from their support group was often the only information they could understand and use. Parents also found that such groups were very valuable in connecting them with other families facing a similar situation. This is supported by previous studies which have reported support groups to facilitate relationship building, skill sharing, decrease parental distress, and increase parents' sense of empowerment and belonging (Davies & Hall, 2005; Grennan et al., 2022; Jackson et al., 2018; Law et al., 2001).

However, despite the informational, practical, and social benefits reported from belonging to a support group, parents' feelings of abandonment by the medical profession and the UK NHS Genetics Service were not eliminated. While the lack of signposting to support groups likely exacerbated the feeling of abandonment, this need for contact with a medical professional did not disappear once parents were settled in a support group. This need for contact with a medical professional has previously been reported by parents for both short‐term (Davies & Hall, 2005) and long‐term support (Oulton et al., 2020). In contrast to the Rare Disease (2016) survey, which found that nearly half (44%) of participants felt that they had another healthcare professional other than their Geneticist to whom they could turn for support, only two people out of 14 in our study (14%) felt that they did. This means that our participants, parents of a child with a very rare or novel DD diagnosis, are a patient group highly in need of additional support post‐diagnosis.

Parents in our study described how the healthcare service is made up of isolated services with no one taking responsibility for their child. Parents felt left to take on the role of coordinating appointments and liaising between medical professionals despite having no medical background and felt medically responsible for their child. These findings were consistent with the findings of a recent study by Oulton et al. (2020) who interviewed and surveyed healthcare professionals and parents of children with an undiagnosed condition who were members of SWAN UK, and found that care coordination was parents' greatest struggle. Moreover, some parents in our study reported how they suffered financially as a result of having to give up paid work to manage this workload of coordinating their child's care, also in keeping with previous studies (Baumusch et al., 2019; Currie & Szabo, 2019; Madeo et al., 2012). Though this support need was consistent with other studies, we are the first study to investigate this in relation to the UK NHS Genetics Service specifically and find that parents often expected that this need for care coordination, oversight in care, and a point of contact with a medical professional would be met by the UK NHS Genetics Service.

Oulton et al. (2020) proposed the need for a new position within the healthcare service, the care coordinator for undiagnosed syndromes. This position was designed to facilitate care coordination and communication between clinicians with the aim of alleviating pressure from parents, challenges also highlighted by participants in our study. This role would fulfill the need for parents to have a single point of contact within the healthcare service, which would reduce feelings of medical abandonment and medical responsibility. Findings from our study support that this role would also benefit those with a diagnosis of a very rare condition.

Overall, support groups and, on occasion, other healthcare professionals, bring both informational and social support which is valuable in closing the gap of unmet support needs. However, parents still felt that they had been abandoned by the medical profession, challenged by care coordination and in need of a point of contact with a medical professional. This gap in support could potentially be met by the role of a care coordinator as outlined by Oulton et al. (2020).

This study also aimed to identify characteristics that indicate those parents that are most in need of additional support. This would allow the healthcare service to target their limited resources to help those in greatest need. This study identified two factors: whether a diagnosis was obtained and whether a partner is present.

Previous research commonly suggests that families without a diagnosis are most in need of additional support due to additional stressors such as lack of information, greater uncertainty, and lack of a healthcare pathway (Madeo et al., 2012; McConkie‐Rosell et al., 2018; Oulton et al., 2020). However, we found that although the lack of a diagnosis did bring these stressors, similar issues and feelings arose for families with a diagnosis where the diagnosis was a very rare or novel disorder. This is supported by the Rare Disease (2016) survey. Furthermore, we found that a main struggle for parents with a child with a DD was coordinating appointments and accessing services regardless of whether a diagnosis was obtained or not. Finally, we found that parents of a child with a very rare or novel diagnosis in particular felt medically abandoned by the UK NHS Genetics Service. This makes sense, as those who are undiagnosed often have multiple appointments as further tests are considered and negative results given. This continued interaction builds relationships and support, almost as a byproduct of the continued diagnostic odyssey. However, once a diagnosis is made, the relationship with the UK NHS Genetics Service usually ends (except when appointments about further pregnancies are requested). We propose that further appointments are of benefit to families, particularly those with novel or ultra‐rare conditions, as their child grows and to review newly published findings about their child's syndrome.

### 5.1 Practice implications

This study suggests the following five recommendations for services to help address these issues:

1. **Set patient expectations and signpost at the point of initial referral**. An initial letter is sent to patients at the point of referral describing the role of the UK NHS Genetics Service, to help set expectations. This letter could also inform patients of the Unique and SWAN UK support groups, enabling families to access these resources and the support available at an early stage.
2. The creation of a **Care Coordinator role**. Increase the number of care coordinators, as outlined by Oulton et al. (2020), to be utilized across the United Kingdom and for these coordinators to be integrated with the UK NHS Genetics Service. The coordinator role should extend to supporting families with a diagnosis of a very rare or novel DD, and potentially all very rare and novel diagnoses where a care pathway or a specialized health care professional is not in place.
3. **Prepare patients for receiving a diagnosis**. Remind patients/parents just prior to their results appointment that the appointment will provide a diagnosis. Inclusion of an additional step in the clinic workflow, whereby patients are informed about the option to bring a support person to the results disclosure, would allow patients to both mentally and practically prepare for a genetic diagnosis. However, this additional step could increase parent anxieties; henceforth, further research into how this could be done is needed.
4. **Increase training for primary care professionals**. Improve training for primary care professionals about both the UK NHS Genetics Service and the main sources of support for children with a DD. This could save parents time, energy, and resources and allow them to access support earlier and prevent negative emotional implications (Julie McMullan et al., 2020).
5. **Increase follow‐up post‐diagnosis**. An automatic follow‐up several weeks post‐diagnosis could be put in place for patients who receive a diagnosis of a very rare or novel diagnosis. Joseph (2019) proposed that the need for follow‐up appointments for diagnostic genetic testing could be met by Genetic Counselors.

The target study group were members of the charities SWAN UK and Unique, who are both UK‐based registered charities. This ascertainment method would omit families who were not members of these groups. The authors recognize that this would have caused some ascertainment bias, but this was deemed the most practical method for recruiting a study cohort with representation from across the whole of the United Kingdom.

### 5.2 Study limitations and further research

The limitations of this study need to be considered when interpreting the results. A limitation of this study was the sampling method of convenience sampling using the support groups Unique and SWAN UK. Studies have shown that participants who choose to enroll in qualitative research studies are most likely to have extreme opinions and experiences; thus, convenience sampling leads to the inclusion of outliers who might be too well represented (Farrokhi, 2012). This would result in biased data, thus reducing how representative the data is of the population sample and resulting in estimating bias (Jager et al., 2017). Furthermore, because the sample was recruited through the SWAN UK and Unique social media pages, the type of participant was restricted to support group members and individuals who have certain skills of using the internet, social media, email, and telephone. Also, this recruited sample will be the most actively engaged support group members who most likely have the most positive views of their support group, further reducing the validity and generalizability of these results. The sample size was also limited due to the time and resources of the authors, meaning more participants could not be recruited, interviewed, and used in analysis. The authors recognize that further sampling could have led to the generation of further themes.

Finally, this study was retrospective. Parents' experiences had sometimes been up to several years before, where some admitted that they struggled to remember their experiences and how they felt at the time. Additionally, some patients referred to the appointments as traumatic and shocking. Trauma and shock have been found to influence memory, meaning that some recollections may not be completely accurate (Vogel & Schwabe, 2016). This may reduce the validity of the results.

In light of this, a quantitative study could validate these results along with identifying characteristics of families that are most in need of additional support. A quantitative study would be able to utilize a much larger sample size to ensure that findings are reliable. As part of this, or separately, research into the role of the clinic coordinator (Oulton et al., 2020) could continue to ensure that the role can help address the unmet support needs of families affected by a DD. Finally, further research into whether a warning just prior to receiving the diagnosis would be helpful and, if so, how this could be done. This research would be explorative due to the lack of previous research on this topic and therefore could be a qualitative study using a sample of both patients and healthcare professionals.

## 6 CONCLUSION

This study highlights the unmet support needs from a medical professional for ongoing care, to be a point of contact, assist in care coordination, and reduce feelings of medical abandonment for parents of a child with a DD with and without a genetic diagnosis. This study suggests that the role of the care coordinator, as outlined by Oulton et al. (2020), could potentially meet these unmet support needs for families affected by a DD. This study also concluded that the UK NHS Genetics Service could do more to manage parents' expectations before their initial appointment by explaining the role and limitations of the Service and addressing the current shortfalls in signposting parents to support groups to further reduce feelings of medical abandonment by the UK NHS Genetics Service.

## AUTHOR CONTRIBUTIONS

The first and second authors confirm that they had full access to all the data in the study and take responsibility for the integrity of the data and the accuracy of the data analysis. All of the authors gave final approval of this version to be published and agree to be accountable for all aspects of the work in ensuring that questions related to the accuracy or integrity of any part of the work are appropriately investigated and resolved.

## CONFLICT OF INTEREST STATEMENT

The authors declare that they have no conflict of interest.

## ETHICS STATEMENT

Human Studies and Informed Consent: The study was approved by the Cardiff University School of Medicine Research Ethics Committee (SMREC). Informed consent was obtained from all patients for being included in the study. All applicable international, national, and/or institutional guidelines were followed.

Animal Studies: No non‐human animal studies were carried out by the authors for this article.

## Supporting information


Data S1:

## Data Availability

The data that support the findings of this study are available from Cardiff University. Restrictions apply to the availability of these data, which were used under license for this study. Data are available from Cardiff University with permission from the first author, Emma Carter (EC), and the University.
