# Supplementary material for: A qualitative study into the experiences of families affected by developmental disorders seeing the UK NHS Genetics Service; if I had known then what I know now
Source: J Genet Couns. 2025 Jun 3;34(3):e70063. doi: 10.1002/jgc4.70063 (PMC12131678; doi:10.1002/jgc4.70063)
Supplement: Supplementary file 1 — Data S1: [file JGC4-34-0-s001.docx]

**Supporting information**

The interview was designed specifically for this study. The topic was researched through an in-depth literature review. Themes and questions were subsequently proposed, discussed, evaluated and condensed by the research team to be left with these questions which were deemed suitable for fulfilling the aims of this study. Demographic and closed questions were included to determine the variability of the sample and to allow comparisons between people based on different factors such as ethnicity, age and socioeconomic status. The interview guide is shown below.

**Interview Guide -** Version 2 28/10/22

Prior to starting the interview, the student researcher will ask the participant if they will audio-recording of the interview and then gain verbal consent to the study. If the participant says no to either, the interview will not go ahead. Once verbal consent is obtained, the student researcher will then say the aims and purpose of the study followed by information on what the role of the clinical genetics department is as written bellow. The interview will then commence.

Aims

1: If parents/ guardians of a child with a DD feel adequately supported by the genetics service, and if not, when and how would they like further support.

2: To explore characteristics of parents/ guardians of a child with a DD that indicate those most in need of further support from the genetics service.

Purpose

This study will fill a gap in the available published evidence, which will be crucial in guiding planning for genetic services.

Role of the Clinical Genetics Department

The role of the Clinical Genetics department is to try and find an underlying cause for a likely genetic condition. Within this, it is the role of this department to answer questions that go along with a genetic diagnosis, for example, what is the chance of another relative/future pregnancy being affected? What is the prognosis for this condition? Why did this happen? Can I be put in touch with other people with the same condition? What treatment options/reproductive options are available? In addition to this, Clinical Genetics can provide a space to discuss the impact a condition has had on an individual/family and allow time for discussion of the implications genetic testing may have. This is known as genetic counselling.

Clinical Genetics typically have appointments that are 30-60 minutes in length and may be given by Clinical Geneticists and/or Genetic Counsellors. They may see families a number of times, but they generally are not a therapeutic counselling service. While Clinical Genetics may guide the management decisions for a condition, they generally are not the professionals who continue this management. Generally, they make referrals to other disciplines for this, for example, Community paediatrics, Cardiology, Neurology etc. However, the aim of this study is to see whether service user expectations are met or if there is a mismatch between expectations and service provision. This could indicate the need for further service developments or increased education of other specialities about the role of the Clinical Genetics service.

Please be aware your answers are confidential and will not be fed back to your genetics service or your support group directly.

Interview questions

1. To which gender do you most identify?
   1. Female
   2. Male
   3. Gender neutral
   4. Transgender female
   5. Transgender male
   6. Other
   7. Prefer not to say
2. What is your ethnic group?
   1. English/Welsh/Scottish/Northern Irish/British
   2. Irish
   3. Gypsy or Irish Traveller
   4. Any other White background, please describe
   5. White and Black Caribbean
   6. White and Black African
   7. White and Asian
   8. Any other Mixed/Multiple ethnic background, please describe
   9. Indian
   10. Pakistani
   11. Bangladeshi
   12. Chinese
   13. Any other Asian background, please describe
   14. African
   15. Caribbean
   16. Any other Black/African/Caribbean background, please describe
   17. Arab
   18. Any other ethnic group, please describe
3. When you saw the Clinical Genetics department, what was the highest level of education you had completed?
   1. No qualifications
   2. High school qualifications or equivalent e.g. GCSC’s
   3. College/ Sixth form qualifications or equivalent e.g. diploma, A-levels, BTEC, NVQ
   4. Bachelor’s degree
   5. Post-graduate degree e.g. Masters, PhD, PGCE
4. What is your relationship with the person with a developmental disorder?
   1. Mother
   2. Father
   3. Sibling
   4. Guardian
   5. Care worker
   6. Other
   7. Prefer not to say
5. What support group did you hear about this study from?
   1. Unique
   2. SWAN
6. Does the affected person have a genetic diagnosis?
   1. Yes
   2. No
7. If you answered yes to question 6, what is the genetic diagnosis?
8. In what region of the UK did you attend your Clinical Genetics appointment?
9. What year was your last appointment with an NHS Clinical Genetics service?
10. How many appointments with the Clinical Genetics service did you attend?
11. When you saw the Clinical Genetics department, how old were you?
    1. 18-25
    2. 26-30
    3. 31-35
    4. 36-40
    5. 41-45
    6. 46-50
    7. 51+
12. When you saw the Clinical Genetics department, how old was the affected person?
    1. 0-3
    2. 4-7
    3. 9-11
    4. 12-15
    5. 15-18
13. When you saw Clinical Genetics, did you feel that you had adequate support from family, a partner/spouse or friends regarding the affected person’s developmental disorder? If yes or no response please expand?
14. What was your overall experience of your interactions with the Clinical Genetic department?
15. Did you feel like you could get back in contact with the Clinical Genetics service since you were discharged if you needed to? Why/why not?
16. Have you felt the need to get back in touch with the Clinical Genetics service since you were seen? If yes, why?
17. Would you say that the genetics professionals met all of your expectations when you saw them? How/ how not?
18. Would you say that the genetics professionals addressed all of your needs when you saw them? How/ how not?
19. If you feel all your needs were not addressed, were they addressed by others e.g. other healthcare specialties, a support group, friends/family – if so, who?
20. Do you have a point of contact for Care Coordination?
21. Is there more the Clinical Genetics department could have done for you/your family? Or would you like them to do more for you/your family in the future?
22. Is there any further information that you would like to share?
